# Supplementary material for: Reliability and utility of blood glucose levels in the periodontal pockets of patients with type 2 diabetes mellitus: a cross-sectional study
Source: PeerJ. 2024 Nov 6;12:e18239. doi: 10.7717/peerj.18239 (PMC11549906; doi:10.7717/peerj.18239)
Supplement: Supplemental Information 2 [file peerj-12-18239-s002.docx]

STROBE Statement—checklist of items that should be included in reports of observational studies

|  | Item No. | Recommendation | Page  No. | Relevant text from manuscript |
| --- | --- | --- | --- | --- |
| **Title and abstract** | 1 | (*a*) Indicate the study’s design with a commonly used term in the title or the abstract | 1 | Reliability and utility of blood glucose levels in the periodontal pockets of patients with type 2 diabetes mellitus: a cross-sectional study. |
|  |  | (*b*) Provide in the abstract an informative and balanced summary of what was done and what was found | 1-2 | Background. Several studies have measured gingival blood glucose (GBG) levels, but few have confirmed systematic bias using Bland–Altman analysis. This study compared the effectiveness of GBG levels with that of fingertip blood glucose (FTBG) levels using Bland–Altman and receiver operating characteristic (ROC) analyses.  Methods. A total of 15 healthy volunteers and 15 patients with type 2 diabetes were selected according to inclusion and exclusion criteria. Each group comprised eight male and seven female participants. The GBG and FTBG levels were measured using a self-monitoring blood glucose device after periodontal examination. Pearson’s product‒moment correlation and simple linear regression analyses were performed. In addition, Bland‒Altman analysis was also performed to assess the degree of agreement between the two methods. ROC analysis was conducted to determine the sensitivity, specificity, and cutoff values for patients with diabetes. The area under the ROC curve (AUC) was used to identify significant differences.  Results. The mean GBG and FTBG levels were 120±44.8 mg/dL and 137±45.1, respectively, for the whole sample. The mean GBG and FTBG levels were 145±47.2 mg/dL and 163±49.1, respectively, in the diabetes group. The mean GBG and FTBG levels in the nondiabetes group were 95.3±25.2 and 111±18.8, respectively. Patients with diabetes were more likely to have a probing pocket depth (PPD) of ≥4 mm at the sampled site. Pearson’s product‒moment correlation and simple linear regression analyses revealed a significant correlation between the GBG and FTBG measurements. Bland–Altman analysis revealed that GBG and FTBG measurements differed significantly among all participants; however, no significant differences were observed among the patients with diabetes (mean difference [MD]±standard deviation [SD]=-18.1±34.2, 95% confidence interval (CI) [-37.0 to 0.88]) or among the participants with a PPD of ≥4 mm (MD±SD=-15.2±30.4, 95% CI [-30.8 to 0.43]). The sensitivity, specificity, and cutoff values of the GBG measurements for detecting diabetes were 80%, 93%, and 123.5 mg/dL, respectively. The sensitivity, specificity, and cutoff values of the FTBG measurements for detecting diabetes were 73%, 87%, and 134.0 mg/dL, respectively. No significant differences were observed between the AUCs (0.078, 95% CI [-0.006 to 0.161]).  Conclusions. The GBG measurements aligned with the FTBG measurements in the patients with diabetes and among the participants with a PPD of ≥4 mm. Patients with diabetes were more likely to have a PPD of ≥4 mm at the sampled site, GBG levels can be used to screen for type 2 diabetes in dental clinics. |
| Introduction | | | |  |
| Background/rationale | 2 | Explain the scientific background and rationale for the investigation being reported | 2-4 | Type 2 diabetes mellitus, characterized by chronic hyperglycemia, can cause complications if not properly treated. Moreover, there is a bidirectional relationship between periodontal disease and diabetes. Patients with diabetes are at high risk of developing periodontal disease and its progression, which in turn affects the progression of diabetes mellitus. Periodontal disease also affects the progression of diabetes mellitus (Graziani et al., 2018). Appropriate periodontal treatment plays an important role in managing patients with diabetes, as periodontal treatment improves insulin resistance and blood glucose management (Simpson et al., 2022). The guidelines for periodontal surgery and tooth extraction recommend that hemoglobin (Hb) A1c levels be maintained at 6.9% for patients with diabetes (Japanese Society of Periodontology, 2014). Furthermore, dental treatment must be postponed if the blood glucose levels are >200 mg/dL or <70 mg/dL (Little et al., 2017). However, measuring blood glucose levels using venous blood sampling is difficult in dental clinics; therefore, dental treatment commences based on physician information. Given the recent relationship between periodontal disease and diabetes, the inability to measure blood glucose levels in the dental outpatient setting is a major problem. Bleeding from gingiva can be a promising resource for measuring blood glucose for dentists as it would be less burden on the patient. If gingival blood glucose measurements are consistent with those of the fingertip, blood glucose measurement from the gingiva can be incorporated into dental blood glucose measurement.  Previous studies on gingival blood glucose (GBG) measurements concluded that GBG levels could be used for diabetes screening (Stein & Nebbia, 1969; Tsutsui, Rich & Schonfeld, 1985). A self-monitoring blood glucose (SMBG) device enables patients to measure their glucose levels by puncturing their fingertips. Several studies have reported strong correlations between fingertip blood glucose (FTBG) levels acquired using an SMBG device and GBG levels (Parker et al., 1993; Beikler et al., 2002; Khader et al., 2006; Ardakani et al., 2009). The measurement of GBG levels is a rapid, safe, noninvasive screening method for diabetes that can be performed during routine periodontal examinations (Parker et al., 1993; Beikler et al., 2002; Khader et al., 2006; Ardakani et al., 2009). Additionally, more patients with diabetes preferred measuring GBG levels over measuring FTBG (Rosedale & Strauss, 2012).  The intraclass correlation coefficient is often used as a measure of reliability to validate new measurement measures. However, what is important in actual measurement is the degree of disagreement. When introducing a new measure, it is necessary to know how much it may differ from previous measures. Since the acceptable measurement error in a clinical setting varies among the types of measurements, no standard can be set automatically by statistical methods (Müller & Behbehani, 2005). Therefore, Bland and Altman reported that an agreement analysis between the two methods was needed (Bland & Altman, 1986; Bland & Altman, 1999).  Bland–Altman analysis has been used to clarify systematic bias (Bland & Altman, 1986; Bland & Altman, 1999). Only two previous studies have reported Bland–Altman analyses of GBG and FTBG levels (Müller & Behbehani, 2004; Müller & Behbehani, 2005; Strauss et al.,2009). Half of the 46 participants in one group did not have periodontitis in the study by Müller & Behbehani, and only 15% had diabetes (Müller & Behbehani, 2004; Müller & Behbehani, 2005). Moreover, Bland–Altman analysis of the GBG and FTBG measurements revealed low concordance between the GBG and FTBG levels (Müller & Behbehani, 2005). The severity of periodontitis was unknown in the study by Strauss et al., and only 9% of the participants had diabetes (Strauss et al., 2009). Bland–Altman analysis revealed adequate agreement between the GBG and FTBG levels in this study (Strauss et al., 2009). Thus, the Bland–Altman analysis results of GBG and FTBG levels have been inconsistent.  To date, several studies have reported that GBG and FTBG values are associated with diabetes and useful for diabetes screening (Suneetha & Rambabu, 2012; Gaikwad et al., 2013; Shetty et al., 2013; Kaur, Singh & Sharma, 2013; Gupta et al., 2014; Dwivedi et al., 2014; Shylaja et al., 2016; Rajesh et al., 2016; Parihar et al., 2016; Partheeban et al., 2017; Sibyl et al., 2017; Mirza et al., 2018; Sande et al., 2020; Rapone et al., 2020; Wu et al., 2021; Patel et al., 2023; Dash et al., 2023; A Alqazlan et al.,2024). Alternatively, other researchers reported that GBG and FTBG values are not associated with diabetes and are not useful for diabetes screening (Debnath et al., 2015; Ansari Moghadam et al., 2024). A recently published systematic review and meta-analysis (Fakheran et al., 2024), citing the three papers mentioned above (Müller & Behbehani, 2004; Müller & Behbehani, 2005; Strauss et al., 2009), reported that GBG values are useful if gingival inflammation is strong and bleeding on probing (BOP) is high. |
| Objectives | 3 | State specific objectives, including any prespecified hypotheses | 4 | This study aimed to evaluate the utility of GBG measurements compared with that of FTBG measurements via Bland–Altman and receiver operating characteristic (ROC) analyses by determining the GBG and FTBG levels in patients with and without diabetes who visited the Division of General Dentistry, Health Sciences University of Hokkaido Hospital (Figure 1). |
| Methods | | | |  |
| Study design | 4 | Present key elements of study design early in the paper | 4 | Inclusion criteria  1. 20 years or older with at least one current tooth undergoing dental treatment at the Division of General Dentistry, Health Sciences University of Hokkaido Hospital.  2. We classified patients with diabetes based on specialists' diabetes diagnoses. Patients in the diabetes group underwent treatment at the Division of Internal Medicine, Health Sciences University of Hokkaido Hospital.  Exclusion criteria  1. Pregnant or lactating women.  2. Patients without diabetes with GBG or FTBG levels greater than 200 mg/dL. |
| Setting | 5 | Describe the setting, locations, and relevant dates, including periods of recruitment, exposure, follow-up, and data collection | 4 | Previous studies were extracted from the PubMed database and examined to determine the number of participants required to evaluate the efficacy of GBG measurements compared with that of FTBG measurements. One study (Rajesh et al., 2016) had 24 participants, one study (Gaikwad et al., 2013) had 25 participants, and three studies (Gupta et al., 2014; Shylaja et al., 2016; Sibyl et al., 2017) with 30 participants reported a significant correlation between the GBG and FTBG measurements. G*Power 3.1 (Faul et al., 2007) obtained from an internet website (Research Team of Cognitive and Industrial Psychology, Department of Experimental Psychology, Faculty of Mathematics and Natural Sciences, Heinrich Heine University Düsseldorf, 2007) was used to calculate the sample size. The α and β errors were set to 0.05 and 0.2, respectively. According to a previous report, a correlation coefficient of 0.715 was selected for the correlation analysis (Gupta et al., 2014). The effect size was set as 0.8 for paired t tests evaluated via Bland–Altman analysis. The sample sizes were calculated as 12 for the correlation analysis and 15 for the paired t test using G*Power 3.1. The total number of participants was 30, with 15 participants each in the diabetic and nondiabetic groups.  Inclusion criteria  1. 20 years or older with at least one current tooth undergoing dental treatment at the Division of General Dentistry, Health Sciences University of Hokkaido Hospital.  2. We classified patients with diabetes based on specialists' diabetes diagnoses. Patients in the diabetes group underwent treatment at the Division of Internal Medicine, Health Sciences University of Hokkaido Hospital.  Exclusion criteria  1. Pregnant or lactating women.  2. Patients without diabetes with GBG or FTBG levels greater than 200 mg/dL.  This study adhered to the tenets of the Declaration of Helsinki. Written informed consent was obtained from the participants after the study was explained. This study was approved by the Ethics Committee of the Institute of Preventive Medical Science, Health Sciences University of Hokkaido (No. 2019_028). |
| Participants | 6 | (*a*) *Cohort study*—Give the eligibility criteria, and the sources and methods of selection of participants. Describe methods of follow-up  *Case-control study*—Give the eligibility criteria, and the sources and methods of case ascertainment and control selection. Give the rationale for the choice of cases and controls  *Cross-sectional study*—Give the eligibility criteria, and the sources and methods of selection of participants | 4 | Inclusion criteria  1. 20 years or older with at least one current tooth undergoing dental treatment at the Division of General Dentistry, Health Sciences University of Hokkaido Hospital.  2. We classified patients with diabetes based on specialists' diabetes diagnoses. Patients in the diabetes group underwent treatment at the Division of Internal Medicine, Health Sciences University of Hokkaido Hospital.  Exclusion criteria  1. Pregnant or lactating women.  2. Patients without diabetes with GBG or FTBG levels greater than 200 mg/dL. |
|  |  | (*b*) *Cohort study*—For matched studies, give matching criteria and number of exposed and unexposed  *Case-control study*—For matched studies, give matching criteria and the number of controls per case | Not applicable | Not applicable |
| Variables | 7 | Clearly define all outcomes, exposures, predictors, potential confounders, and effect modifiers. Give diagnostic criteria, if applicable | 4-5 | Medical and dental examinations  Details regarding sex, age, the time at which the participants finished their last meal on the day of examination, whether they had visited a physician, and smoking history were recorded. In addition, the most recent HbA1c levels were also recorded, and patients with type 2 diabetes were assigned to the diabetes group.  Multiple examiners conducted periodontal examinations, and an interexaminer reliability assessment was conducted. Dental examinations were subsequently performed. The number of teeth present was recorded. In addition, the dental plaque was stained, and the plaque control record (PCR) was determined (O'Leary, Drake & Naylor, 1972). The probing pocket depth (PPD) and BOP were evaluated using a periodontal pocket probe (CP-11, Hu-Friedy, Chicago, IL, USA) and recorded. Tooth mobility was measured with dental tweezers using Miller’s mobility index (Japanese Society of Periodontology, 2017; Wu et al., 2018) and classified as follows: Grade 0 (physiological mobility), ≤0.2 mm; Grade 1 (slight, labiolingual), 0.2–1 mm; Grade 2 (moderate, labiolingual and mesiodistal), 1–2 mm; and Grade 3 (severe, labiolingual and mesiodistal) ≥2 mm or vertical movement. The periodontal inflamed surface area (PISA) and periodontal epithelial surface area (PESA) were calculated (Nesse et al., 2008) using a spreadsheet (Vissink et al., 2008a) available on an internet website (Vissink et al., 2008b), and the PPD and BOP were entered. Periodontitis was diagnosed and classified based on the examinations and radiographs (Tonetti, Greenwell & Kornman, 2018a; Tonetti, Greenwell & Kornman, 2018b). Stages indicating the severity and complexity of periodontitis were as follows: Stage I, initial; Stage II, moderate; Stage III, severe with potential for additional tooth loss; and Stage IV, severe with potential for loss of dentition. The extent was defined as generalized (>30% of teeth involved) or localized for each stage. In addition, the grade, which indicates the risk of periodontitis progression, was defined as follows: Grade A, slow rate of progression; Grade B, moderate rate of progression; and Grade C, rapid rate of progression. Risk factors, including smoking and diabetes, were considered when the grade was determined.  Blood glucose measurements  The dental plaque was gently removed with cotton pellets, and the sampling sites were isolated using cotton rolls to prevent contamination with saliva and dental plaque. A saliva ejector was also used. Periodontal probing of the sampling sites was subsequently performed. A micropipette (Eppendorf Reference 2, Eppendorf AG, Hamburg, Germany) collected approximately 1.0 μL (the minimum volume required for a single blood glucose level measurement) of blood from the sampling site. The mandibular sampling sites were restricted to the labial or buccal sides of the teeth to prevent salivary contamination. The labial or palatal sides of the maxillary anterior teeth were sampled, whereas the palatal sides of the maxillary premolars or molars were sampled. Periodontal pockets with pus were excluded. Blood samples were also acquired from the fingertip. The blood glucose levels were measured using an SMBG device (FreeStyle Precision Neo, Abbott Diabetes Care, Inc., Alameda, CA, USA) immediately after blood collection. |
| Data sources/ measurement | 8* | For each variable of interest, give sources of data and details of methods of assessment (measurement). Describe comparability of assessment methods if there is more than one group | 4-5 | Medical and dental examinations  Details regarding sex, age, the time at which the participants finished their last meal on the day of examination, whether they had visited a physician, and smoking history were recorded. In addition, the most recent HbA1c levels were also recorded, and patients with type 2 diabetes were assigned to the diabetes group.  Multiple examiners conducted periodontal examinations, and an interexaminer reliability assessment was conducted. Dental examinations were subsequently performed. The number of teeth present was recorded. In addition, the dental plaque was stained, and the plaque control record (PCR) was determined (O'Leary, Drake & Naylor, 1972). The probing pocket depth (PPD) and BOP were evaluated using a periodontal pocket probe (CP-11, Hu-Friedy, Chicago, IL, USA) and recorded. Tooth mobility was measured with dental tweezers using Miller’s mobility index (Japanese Society of Periodontology, 2017; Wu et al., 2018) and classified as follows: Grade 0 (physiological mobility), ≤0.2 mm; Grade 1 (slight, labiolingual), 0.2–1 mm; Grade 2 (moderate, labiolingual and mesiodistal), 1–2 mm; and Grade 3 (severe, labiolingual and mesiodistal) ≥2 mm or vertical movement. The periodontal inflamed surface area (PISA) and periodontal epithelial surface area (PESA) were calculated (Nesse et al., 2008) using a spreadsheet (Vissink et al., 2008a) available on an internet website (Vissink et al., 2008b), and the PPD and BOP were entered. Periodontitis was diagnosed and classified based on the examinations and radiographs (Tonetti, Greenwell & Kornman, 2018a; Tonetti, Greenwell & Kornman, 2018b). Stages indicating the severity and complexity of periodontitis were as follows: Stage I, initial; Stage II, moderate; Stage III, severe with potential for additional tooth loss; and Stage IV, severe with potential for loss of dentition. The extent was defined as generalized (>30% of teeth involved) or localized for each stage. In addition, the grade, which indicates the risk of periodontitis progression, was defined as follows: Grade A, slow rate of progression; Grade B, moderate rate of progression; and Grade C, rapid rate of progression. Risk factors, including smoking and diabetes, were considered when the grade was determined.  Blood glucose measurements  The dental plaque was gently removed with cotton pellets, and the sampling sites were isolated using cotton rolls to prevent contamination with saliva and dental plaque. A saliva ejector was also used. Periodontal probing of the sampling sites was subsequently performed. A micropipette (Eppendorf Reference 2, Eppendorf AG, Hamburg, Germany) collected approximately 1.0 μL (the minimum volume required for a single blood glucose level measurement) of blood from the sampling site. The mandibular sampling sites were restricted to the labial or buccal sides of the teeth to prevent salivary contamination. The labial or palatal sides of the maxillary anterior teeth were sampled, whereas the palatal sides of the maxillary premolars or molars were sampled. Periodontal pockets with pus were excluded. Blood samples were also acquired from the fingertip. The blood glucose levels were measured using an SMBG device (FreeStyle Precision Neo, Abbott Diabetes Care, Inc., Alameda, CA, USA) immediately after blood collection. |
| Bias | 9 | Describe any efforts to address potential sources of bias | 4-5 | Sample size setting  Previous studies were extracted from the PubMed database and examined to determine the number of participants required to evaluate the efficacy of GBG measurements compared with that of FTBG measurements. One study (Rajesh et al., 2016) had 24 participants, one study (Gaikwad et al., 2013) had 25 participants, and three studies (Gupta et al., 2014; Shylaja et al., 2016; Sibyl et al., 2017) with 30 participants reported a significant correlation between the GBG and FTBG measurements. G*Power 3.1 (Faul et al., 2007) obtained from an internet website (Research Team of Cognitive and Industrial Psychology, Department of Experimental Psychology, Faculty of Mathematics and Natural Sciences, Heinrich Heine University Düsseldorf, 2007) was used to calculate the sample size. The α and β errors were set to 0.05 and 0.2, respectively. According to a previous report, a correlation coefficient of 0.715 was selected for the correlation analysis (Gupta et al., 2014). The effect size was set as 0.8 for paired t tests evaluated via Bland–Altman analysis. The sample sizes were calculated as 12 for the correlation analysis and 15 for the paired t test using G*Power 3.1. The total number of participants was 30, with 15 participants each in the diabetic and nondiabetic groups.  Inclusion criteria  1. 20 years or older with at least one current tooth undergoing dental treatment at the Division of General Dentistry, Health Sciences University of Hokkaido Hospital.  2. We classified patients with diabetes based on specialists' diabetes diagnoses. Patients in the diabetes group underwent treatment at the Division of Internal Medicine, Health Sciences University of Hokkaido Hospital.  Exclusion criteria  1. Pregnant or lactating women.  2. Patients without diabetes with GBG or FTBG levels greater than 200 mg/dL.  This study adhered to the tenets of the Declaration of Helsinki. Written informed consent was obtained from the participants after the study was explained. This study was approved by the Ethics Committee of the Institute of Preventive Medical Science, Health Sciences University of Hokkaido (No. 2019_028).  Medical and dental examinations  Details regarding sex, age, the time at which the participants finished their last meal on the day of examination, whether they had visited a physician, and smoking history were recorded. In addition, the most recent HbA1c levels were also recorded, and patients with type 2 diabetes were assigned to the diabetes group.  Multiple examiners conducted periodontal examinations, and an interexaminer reliability assessment was conducted. Dental examinations were subsequently performed. The number of teeth present was recorded. In addition, the dental plaque was stained, and the plaque control record (PCR) was determined (O'Leary, Drake & Naylor, 1972). The probing pocket depth (PPD) and BOP were evaluated using a periodontal pocket probe (CP-11, Hu-Friedy, Chicago, IL, USA) and recorded. Tooth mobility was measured with dental tweezers using Miller’s mobility index (Japanese Society of Periodontology, 2017; Wu et al., 2018) and classified as follows: Grade 0 (physiological mobility), ≤0.2 mm; Grade 1 (slight, labiolingual), 0.2–1 mm; Grade 2 (moderate, labiolingual and mesiodistal), 1–2 mm; and Grade 3 (severe, labiolingual and mesiodistal) ≥2 mm or vertical movement. The periodontal inflamed surface area (PISA) and periodontal epithelial surface area (PESA) were calculated (Nesse et al., 2008) using a spreadsheet (Vissink et al., 2008a) available on an internet website (Vissink et al., 2008b), and the PPD and BOP were entered. Periodontitis was diagnosed and classified based on the examinations and radiographs (Tonetti, Greenwell & Kornman, 2018a; Tonetti, Greenwell & Kornman, 2018b). Stages indicating the severity and complexity of periodontitis were as follows: Stage I, initial; Stage II, moderate; Stage III, severe with potential for additional tooth loss; and Stage IV, severe with potential for loss of dentition. The extent was defined as generalized (>30% of teeth involved) or localized for each stage. In addition, the grade, which indicates the risk of periodontitis progression, was defined as follows: Grade A, slow rate of progression; Grade B, moderate rate of progression; and Grade C, rapid rate of progression. Risk factors, including smoking and diabetes, were considered when the grade was determined.  Blood glucose measurements  The dental plaque was gently removed with cotton pellets, and the sampling sites were isolated using cotton rolls to prevent contamination with saliva and dental plaque. A saliva ejector was also used. Periodontal probing of the sampling sites was subsequently performed. A micropipette (Eppendorf Reference 2, Eppendorf AG, Hamburg, Germany) collected approximately 1.0 μL (the minimum volume required for a single blood glucose level measurement) of blood from the sampling site. The mandibular sampling sites were restricted to the labial or buccal sides of the teeth to prevent salivary contamination. The labial or palatal sides of the maxillary anterior teeth were sampled, whereas the palatal sides of the maxillary premolars or molars were sampled. Periodontal pockets with pus were excluded. Blood samples were also acquired from the fingertip. The blood glucose levels were measured using an SMBG device (FreeStyle Precision Neo, Abbott Diabetes Care, Inc., Alameda, CA, USA) immediately after blood collection. |
| Study size | 10 | Explain how the study size was arrived at | 4 | Sample size setting  Previous studies were extracted from the PubMed database and examined to determine the number of participants required to evaluate the efficacy of GBG measurements compared with that of FTBG measurements. One study (Rajesh et al., 2016) had 24 participants, one study (Gaikwad et al., 2013) had 25 participants, and three studies (Gupta et al., 2014; Shylaja et al., 2016; Sibyl et al., 2017) with 30 participants reported a significant correlation between the GBG and FTBG measurements. G*Power 3.1 (Faul et al., 2007) obtained from an internet website (Research Team of Cognitive and Industrial Psychology, Department of Experimental Psychology, Faculty of Mathematics and Natural Sciences, Heinrich Heine University Düsseldorf, 2007) was used to calculate the sample size. The α and β errors were set to 0.05 and 0.2, respectively. According to a previous report, a correlation coefficient of 0.715 was selected for the correlation analysis (Gupta et al., 2014). The effect size was set as 0.8 for paired t tests evaluated via Bland–Altman analysis. The sample sizes were calculated as 12 for the correlation analysis and 15 for the paired t test using G*Power 3.1. The total number of participants was 30, with 15 participants each in the diabetic and nondiabetic groups.  Inclusion criteria  1. 20 years or older with at least one current tooth undergoing dental treatment at the Division of General Dentistry, Health Sciences University of Hokkaido Hospital.  2. We classified patients with diabetes based on specialists' diabetes diagnoses. Patients in the diabetes group underwent treatment at the Division of Internal Medicine, Health Sciences University of Hokkaido Hospital.  Exclusion criteria  1. Pregnant or lactating women.  2. Patients without diabetes with GBG or FTBG levels greater than 200 mg/dL.  This study adhered to the tenets of the Declaration of Helsinki. Written informed consent was obtained from the participants after the study was explained. This study was approved by the Ethics Committee of the Institute of Preventive Medical Science, Health Sciences University of Hokkaido (No. 2019_028). |

Continued on next page

| Quantitative variables | 11 | Explain how quantitative variables were handled in the analyses. If applicable, describe which groupings were chosen and why | 5-7 | Clinical characteristics of the participants  All the statistical analyses were performed via SPSS Statistics Version 26 (IBM, Chicago, IL, USA). Categorical variables of the diabetic and nondiabetic groups were analyzed according to sex, smoking habits, diagnosis and classification of periodontitis, and PPDs of ≥4 mm and ≤3 mm at the blood sampling site. The chi-square or Fisher's exact tests were used to examine these parameters. Age and HbA1c levels in the diabetic group, blood glucose levels, and postprandial time were continuous variables. The continuous dental variables were the number of teeth present, PPD, BOP, PISA, PESA, PCR, and tooth mobility. The mean ± standard deviation values were calculated. Continuous variables of the diabetic and nondiabetic groups were analyzed using a two-sample t tests.  Correlation and regression analyses  The correlation between the GBG and FTBG measurements was analyzed to determine whether the GBG measurements were as reliable as the FTBG measurements. The Pearson's product‒moment correlation coefficient (r) was used to evaluate the correlation. In addition, the simple linear regression equation {FTBG = constant + simple linear regression coefficient (R) × GBG} and the coefficient of determination (R²) for simple linear regression analysis were determined (Müller & Behbehani, 2005; Rajesh et al., 2016). In the case of simple linear regression analysis, there is only one explanatory variable, so the R is equal to the r.  Bland–Altman analysis  The Bland–Altman analysis was performed as follows (Bland & Altman, 1986; Bland & Altman, 1999; Müller & Behbehani, 2005; Strauss et al., 2009; IBM Support, 2020). The interpretation of bias was based on the mean difference (MD) between the GBG and FTBG values. Bias and MD are synonymous. The limit of agreement (LOA) is the range corresponding to bias (MD) ±1.96×standard deviation (SD) for the 95% confidence interval (CI), and theoretically, 95% of the differences in the measured values converge to the LOA. The MD ± SD between GBG and FTBG and the 95% confidence interval (95% CI) of the MD were calculated. A one-sample t test was then used to compare GBG and FTBG, and a fixed bias was considered present if the 95% CI did not exceed zero. The coefficient of agreement (COA) and LOAs were determined. The COA was calculated as 1.96 × SD. The LOA was calculated as the MD±COA. The 95% CIs of the upper and lower LOAs were also calculated. A simple linear regression analysis was performed with the difference between GBG and FTBG as the objective variable and (GBG+FTBG)/2 as the explanatory variable to determine the presence of proportional bias, and the significance was tested. Proportional bias was considered present if the R was judged to be significant. Furthermore, only the MD ± SD and evaluation up to 95% CIs were used to determine fixed bias in the presence of fixed and proportional bias. Sites with a PPD of ≥4 mm were considered suitable for obtaining the same GBG value as the FTBG value (Strauss et al., 2009). A subgroup analysis for PPDs of ≥4 mm and ≤3 mm was also conducted.  Receiver operating characteristic analysis  The ROC curves of the GBG and FTBG levels were plotted, and the areas under the curves (AUCs) were calculated. The sensitivity and specificity were also calculated. The optimal cutoff values for diabetes screening were subsequently determined. The cutoff values were calculated as the coordinate point, i.e., the minimum value of (1-sensitivity) ² + (1-specificity) ² from the coordinate point table of the ROC curve (Strauss et al., 2012; Strauss et al., 2015). The difference between the AUCs was determined via a one-sample t test if the AUCs of the GBG and FTBG values were significant. All significance levels were set at p<0.05. |
| --- | --- | --- | --- | --- |
| Statistical methods | 12 | (*a*) Describe all statistical methods, including those used to control for confounding | 5-7 | Clinical characteristics of the participants  All the statistical analyses were performed via SPSS Statistics Version 26 (IBM, Chicago, IL, USA). Categorical variables of the diabetic and nondiabetic groups were analyzed according to sex, smoking habits, diagnosis and classification of periodontitis, and PPDs of ≥4 mm and ≤3 mm at the blood sampling site. The chi-square or Fisher's exact tests were used to examine these parameters. Age and HbA1c levels in the diabetic group, blood glucose levels, and postprandial time were continuous variables. The continuous dental variables were the number of teeth present, PPD, BOP, PISA, PESA, PCR, and tooth mobility. The mean ± standard deviation values were calculated. Continuous variables of the diabetic and nondiabetic groups were analyzed using a two-sample t tests.  Correlation and regression analyses  The correlation between the GBG and FTBG measurements was analyzed to determine whether the GBG measurements were as reliable as the FTBG measurements. The Pearson's product‒moment correlation coefficient (r) was used to evaluate the correlation. In addition, the simple linear regression equation {FTBG = constant + simple linear regression coefficient (R) × GBG} and the coefficient of determination (R²) for simple linear regression analysis were determined (Müller & Behbehani, 2005; Rajesh et al., 2016). In the case of simple linear regression analysis, there is only one explanatory variable, so the R is equal to the r.  Bland–Altman analysis  The Bland–Altman analysis was performed as follows (Bland & Altman, 1986; Bland & Altman, 1999; Müller & Behbehani, 2005; Strauss et al., 2009; IBM Support, 2020). The interpretation of bias was based on the mean difference (MD) between the GBG and FTBG values. Bias and MD are synonymous. The limit of agreement (LOA) is the range corresponding to bias (MD) ±1.96×standard deviation (SD) for the 95% confidence interval (CI), and theoretically, 95% of the differences in the measured values converge to the LOA. The MD ± SD between GBG and FTBG and the 95% confidence interval (95% CI) of the MD were calculated. A one-sample t test was then used to compare GBG and FTBG, and a fixed bias was considered present if the 95% CI did not exceed zero. The coefficient of agreement (COA) and LOAs were determined. The COA was calculated as 1.96 × SD. The LOA was calculated as the MD±COA. The 95% CIs of the upper and lower LOAs were also calculated. A simple linear regression analysis was performed with the difference between GBG and FTBG as the objective variable and (GBG+FTBG)/2 as the explanatory variable to determine the presence of proportional bias, and the significance was tested. Proportional bias was considered present if the R was judged to be significant. Furthermore, only the MD ± SD and evaluation up to 95% CIs were used to determine fixed bias in the presence of fixed and proportional bias. Sites with a PPD of ≥4 mm were considered suitable for obtaining the same GBG value as the FTBG value (Strauss et al., 2009). A subgroup analysis for PPDs of ≥4 mm and ≤3 mm was also conducted.  Receiver operating characteristic analysis  The ROC curves of the GBG and FTBG levels were plotted, and the areas under the curves (AUCs) were calculated. The sensitivity and specificity were also calculated. The optimal cutoff values for diabetes screening were subsequently determined. The cutoff values were calculated as the coordinate point, i.e., the minimum value of (1-sensitivity) ² + (1-specificity) ² from the coordinate point table of the ROC curve (Strauss et al., 2012; Strauss et al., 2015). The difference between the AUCs was determined via a one-sample t test if the AUCs of the GBG and FTBG values were significant. All significance levels were set at p<0.05. |
|  |  | (*b*) Describe any methods used to examine subgroups and interactions | 6 | Correlation and regression analyses  The correlation between the GBG and FTBG measurements was analyzed to determine whether the GBG measurements were as reliable as the FTBG measurements. The Pearson's product‒moment correlation coefficient (r) was used to evaluate the correlation. In addition, the simple linear regression equation {FTBG = constant + simple linear regression coefficient (R) × GBG} and the coefficient of determination (R²) for simple linear regression analysis were determined (Müller & Behbehani, 2005; Rajesh et al., 2016). In the case of simple linear regression analysis, there is only one explanatory variable, so the R is equal to the r.  Bland–Altman analysis  The Bland–Altman analysis was performed as follows (Bland & Altman, 1986; Bland & Altman, 1999; Müller & Behbehani, 2005; Strauss et al., 2009; IBM Support, 2020). The interpretation of bias was based on the mean difference (MD) between the GBG and FTBG values. Bias and MD are synonymous. The limit of agreement (LOA) is the range corresponding to bias (MD) ±1.96×standard deviation (SD) for the 95% confidence interval (CI), and theoretically, 95% of the differences in the measured values converge to the LOA. The MD ± SD between GBG and FTBG and the 95% confidence interval (95% CI) of the MD were calculated. A one-sample t test was then used to compare GBG and FTBG, and a fixed bias was considered present if the 95% CI did not exceed zero. The coefficient of agreement (COA) and LOAs were determined. The COA was calculated as 1.96 × SD. The LOA was calculated as the MD±COA. The 95% CIs of the upper and lower LOAs were also calculated. A simple linear regression analysis was performed with the difference between GBG and FTBG as the objective variable and (GBG+FTBG)/2 as the explanatory variable to determine the presence of proportional bias, and the significance was tested. Proportional bias was considered present if the R was judged to be significant. Furthermore, only the MD ± SD and evaluation up to 95% CIs were used to determine fixed bias in the presence of fixed and proportional bias. Sites with a PPD of ≥4 mm were considered suitable for obtaining the same GBG value as the FTBG value (Strauss et al., 2009). A subgroup analysis for PPDs of ≥4 mm and ≤3 mm was also conducted. |
|  |  | (*c*) Explain how missing data were addressed | Not applicable | Not applicable |
|  |  | (*d*) *Cohort study*—If applicable, explain how loss to follow-up was addressed  *Case-control study*—If applicable, explain how matching of cases and controls was addressed  *Cross-sectional study*—If applicable, describe analytical methods taking account of sampling strategy | Not applicable | Not applicable |
|  |  | (*e*) Describe any sensitivity analyses | 6-7 | Receiver operating characteristic analysis  The ROC curves of the GBG and FTBG levels were plotted, and the areas under the curves (AUCs) were calculated. The sensitivity and specificity were also calculated. The optimal cutoff values for diabetes screening were subsequently determined. The cutoff values were calculated as the coordinate point, i.e., the minimum value of (1-sensitivity) ² + (1-specificity) ² from the coordinate point table of the ROC curve (Strauss et al., 2012; Strauss et al., 2015). The difference between the AUCs was determined via a one-sample t test if the AUCs of the GBG and FTBG values were significant. |
| Results | | | | |
| Participants | 13* | (a) Report numbers of individuals at each stage of study—eg numbers potentially eligible, examined for eligibility, confirmed eligible, included in the study, completing follow-up, and analysed | 6 | Informed consent was obtained from 37 participants between November 2020 and June 2021. The diabetic and nondiabetic groups comprised 15 and 22 participants, respectively. Therefore, case‒control matching was performed in the diabetic and nondiabetic groups for 15 participants. Eight male and seven female participants matched perfectly. The analysis was conducted under these conditions, as the minimum age difference possible was within 8 years. The supplemental file (Data S1) presents the raw data before analysis. |
|  |  | (b) Give reasons for non-participation at each stage | Not applicable | Not applicable |
|  |  | (c) Consider use of a flow diagram | Not applicable | Not applicable |
| Descriptive data | 14* | (a) Give characteristics of study participants (eg demographic, clinical, social) and information on exposures and potential confounders | 7 | The diabetic group did not include patients with Grade A periodontitis; in contrast, 14 of the 15 participants (93%) in the nondiabetic group had Grade A or B periodontitis (Table 1), indicating a significant difference (p=0.001). The participants in the diabetic group were significantly more likely to have generalized chronic periodontitis (14 of the 15 patients, 93%) (p=0.040). The mean number of present teeth was 19.9±4.85 and 23.7±3.81 in the diabetic and nondiabetic groups, respectively, indicating a significant difference (p=0.026; Table 2). |
|  |  | (b) Indicate number of participants with missing data for each variable of interest | Not applicable | Not applicable |
|  |  | (c) *Cohort study*—Summarise follow-up time (eg, average and total amount) | Not applicable | Not applicable |
| Outcome data | 15* | *Cohort study*—Report numbers of outcome events or summary measures over time | Not applicable | Not applicable |
|  |  | *Case-control study—*Report numbers in each exposure category, or summary measures of exposure | Not applicable | Not applicable |
|  |  | *Cross-sectional study—*Report numbers of outcome events or summary measures | 7 | The diabetic group did not include patients with Grade A periodontitis; in contrast, 14 of the 15 participants (93%) in the nondiabetic group had Grade A or B periodontitis (Table 1), indicating a significant difference (p=0.001). The participants in the diabetic group were significantly more likely to have generalized chronic periodontitis (14 of the 15 patients, 93%) (p=0.040). The mean number of present teeth was 19.9±4.85 and 23.7±3.81 in the diabetic and nondiabetic groups, respectively, indicating a significant difference (p=0.026; Table 2). |
| Main results | 16 | (*a*) Give unadjusted estimates and, if applicable, confounder-adjusted estimates and their precision (eg, 95% confidence interval). Make clear which confounders were adjusted for and why they were included | 7-8 | Figure 2 shows the correlation and simple linear regression between the GBG and FTBG levels in all participants. The correlation coefficient and the simple linear regression equation were r=0.827 (p<0.001) and FTBG value=36.970+0.827×GBG value (95% CI for R=0.613 to 1.051, p<0.001), R²=0.684, respectively.  The Bland–Altman analysis revealed a significant difference in fixed bias (MD±SD=-16.8±26.4 mg/dL, 95% CI for MD=-26.7 to -6.94 mg/dL, p=0.002; Fig. 3 and Table 3). The LOA ranged from -68.6 to 35.0 mg/dL. Proportional bias was considered nil, as no significant difference was observed (R=-0.006, 95% CI for R=-0.244 to 0.232, p=0.959).  Figure 4 presents the correlation and simple linear regression between the GBG and FTBG levels in the nondiabetic group. The correlation coefficient and the simple linear regression equation were r=0.756 (p<0.001) and FTBG value=57.028+0.756×GBG value (95% CI for R=0.272 to 0.858, p=0.001), R²=0.572, respectively.  The Bland–Altman analysis revealed a significant difference in fixed bias among the participants without diabetes (MD±SD=-15.5±16.5 mg/dL, 95% CI for MD=-24.7 to -6.41 mg/dL, p=0.003; Fig. 5 and Table 3). LOA ranged from -47.8 to 16.8 mg/dL. Proportional bias was considered nil, as no significant difference was observed (R=0.329, 95% CI for R=-0.107 to 0.765, p=0.127).  Figure 6 depicts the correlation and simple linear regression between the GBG and FTBG levels in the diabetic group. The correlation coefficient and the simple linear regression equation were r=0.748 (p<0.010) and FTBG value = 49.966 + 0.748×GBG value (95% CI for R=0.365 to 1.194, p=0.001), R²=0.560, respectively.  The Bland–Altman analysis revealed no significant difference in fixed bias among the participants with diabetes (MD±SD=-18.1±34.2 mg/dL, 95% CI for MD=-37.0 to 0.88 mg/dL, p=0.060; Fig. 7 and Table 3). The LOA ranged from -85.1 to 49.0 mg/dL. Proportional bias was considered nil, as no significant difference was observed (R=-0.047, 95% CI for R=-0.501 to 0.408, p=0.827). |
|  |  | (*b*) Report category boundaries when continuous variables were categorized | 8 | Figure 8 depicts the correlation and simple linear regression between the GBG and FTBG levels in the subgroups with a PPD of ≥4 mm at the GBG sampling sites (n=17). The correlation coefficient and the simple linear regression equation were r=0.838 (p<0.001) and FTBG value=43.750+0.838×GBG value (95% CI for R=0.499 to 1.055, p<0.001), R²=0.572, respectively, which is significant (Fig. 8).  The Bland–Altman analysis of the PPD ≥4 mm subgroup revealed no significant differences in fixed bias (MD±SD=-15.2±30.4 mg/dL, 95% CI for MD=-30.8 to 0.43 mg/dL, p=0.056; Fig. 9 and Table 4). LOA ranged from -74.7 to 44.3 mg/dL. Proportional bias was considered nil, as no significant difference was observed (R=-0.082, 95% CI for R=-0.244 to 0.408, p=0.599).  Figure 10 depicts the correlation and simple linear regression between the GBG and FTBG levels in the PPD ≤3 mm subgroup (n=13). The correlation coefficient and the simple linear regression equation were r=0.823 (p<0.001) and FTBG value = -2.804 + 0.823×GBG value (95% CI for R=0.650 to 1.748, p=0.001), R²=0.677, respectively (Fig. 10).  The Bland–Altman analysis of the PPD ≤3 mm subgroup revealed a significant difference in fixed bias (MD±SD=-18.9±21.2 mg/dL, 95% CI for MD=-31.7 to -6.11 mg/dL, p=0.007; Fig. 11 and Table 4). Proportional bias was considered significant (R=-0.774, 95% CI for R=-1.533 to -0.016, p=0.046). |
|  |  | (*c*) If relevant, consider translating estimates of relative risk into absolute risk for a meaningful time period | Not applicable | Not applicable |

Continued on next page

| Other analyses | 17 | Report other analyses done—eg analyses of subgroups and interactions, and sensitivity analyses | 8 | Figure 8 depicts the correlation and simple linear regression between the GBG and FTBG levels in the subgroups with a PPD of ≥4 mm at the GBG sampling sites (n=17). The correlation coefficient and the simple linear regression equation were r=0.838 (p<0.001) and FTBG value=43.750+0.838×GBG value (95% CI for R=0.499 to 1.055, p<0.001), R²=0.572, respectively, which is significant (Fig. 8).  The Bland–Altman analysis of the PPD ≥4 mm subgroup revealed no significant differences in fixed bias (MD±SD=-15.2±30.4 mg/dL, 95% CI for MD=-30.8 to 0.43 mg/dL, p=0.056; Fig. 9 and Table 4). LOA ranged from -74.7 to 44.3 mg/dL. Proportional bias was considered nil, as no significant difference was observed (R=-0.082, 95% CI for R=-0.244 to 0.408, p=0.599).  Figure 10 depicts the correlation and simple linear regression between the GBG and FTBG levels in the PPD ≤3 mm subgroup (n=13). The correlation coefficient and the simple linear regression equation were r=0.823 (p<0.001) and FTBG value = -2.804 + 0.823×GBG value (95% CI for R=0.650 to 1.748, p=0.001), R²=0.677, respectively (Fig. 10).  The Bland–Altman analysis of the PPD ≤3 mm subgroup revealed a significant difference in fixed bias (MD±SD=-18.9±21.2 mg/dL, 95% CI for MD=-31.7 to -6.11 mg/dL, p=0.007; Fig. 11 and Table 4). Proportional bias was considered significant (R=-0.774, 95% CI for R=-1.533 to -0.016, p=0.046).  The AUC of the GBG value was 0.880 (95% CI=0.752 to 1.000, p<0.001; Fig. 12). The AUC of the FTBG values was 0.802 (95% CI=0.641 to 0.963, p=0.005). The sensitivity, specificity, and cutoff values were obtained, as both AUC values were significant. The sensitivity, specificity, and cutoff values of the GBG values were 0.800, 0.933, and 123.5 mg/dL, respectively (Table 5). The sensitivity, specificity, and cutoff values of the FTBG values were 0.733, 0.867, and 134.0 mg/dL, respectively (Table 6). The significant difference in AUCs between the GBG and FTBG levels was tested. The difference between the AUCs of the GBG and FTBG values was 0.078 (95% CI=-0.006 to 0.161, p=0.068), which was not significant (Fig. 12). |
| --- | --- | --- | --- | --- |
| Discussion | | | | |
| Key results | 18 | Summarise key results with reference to study objectives | 8-9 | The Bland–Altman analysis revealed no significant difference between GBG and FTBG levels in the diabetes or PPD ≥4 mm groups. Similarly, a Bland–Altman analysis revealed no significant difference between the GBG and FTBG levels in a group with PPD ≥4 mm in a previous study (Strauss et al., 2009).  The sensitivity, specificity, and cutoff value of the GBG measurements for detecting diabetes were 80%, 93%, and 123.5 mg/dL, respectively. The sensitivity, specificity, and cutoff value of the FTBG measurements for detecting diabetes were 73%, 87%, and 134.0 mg/dL, respectively. No significant differences were observed between the AUCs (0.078, 95% CI [-0.006 to -0.161]). |
| Limitations | 19 | Discuss limitations of the study, taking into account sources of potential bias or imprecision. Discuss both direction and magnitude of any potential bias | 9 | Differences in GBG due to periodontitis status should be explored in future studies. Furthermore, the sample size of 15 participants per group might limit the generalizability of the findings. Future studies with larger sample sizes are necessary to validate these results. |
| Interpretation | 20 | Give a cautious overall interpretation of results considering objectives, limitations, multiplicity of analyses, results from similar studies, and other relevant evidence | 9-10 | This study hypothesizes that GBG measurements may be an effective screening tool for type 2 diabetes mellitus, as periodontitis tends to be more severe, and BOP tends to be greater in patients with type 2 diabetes mellitus. The GBG and FTBG levels were reliable in the diabetic group and patients with a PPD ≥4 mm. Furthermore, the sensitivity and specificity were as high as those reported in previous studies. Therefore, random blood glucose levels measured from periodontal pockets of patients with diabetes and patients with periodontitis with a PPD of ≥4 mm may be useful for screening for type 2 diabetes mellitus. These findings are expected to contribute to the early detection of type 2 diabetes through the dental setting.  The limitation of this study is a small sample size that cannot allow us to current data as a standard for the general population. Although measurement using Bland-Altman, correlation, and regression analyses determined the usefulness of the GBG, however, more accurate measurements need to be confirmed using a large number of participants with a variety of clinical conditions.  This study suggests that GBG may be useful for screening for diabetes in dentistry. In contrast, there may be limitations regarding the degree of concordance with FTBG. Local factors such as inflammatory cytokines in periodontal tissues may influence GBG, and further investigation of these factors may allow GBG to be applied in diagnosing and treating diabetes-related periodontitis. |
| Generalisability | 21 | Discuss the generalisability (external validity) of the study results | 9-10 | This study hypothesizes that GBG measurements may be an effective screening tool for type 2 diabetes mellitus, as periodontitis tends to be more severe, and BOP tends to be greater in patients with type 2 diabetes mellitus. The GBG and FTBG levels were reliable in the diabetic group and patients with a PPD ≥4 mm. Furthermore, the sensitivity and specificity were as high as those reported in previous studies. Therefore, random blood glucose levels measured from periodontal pockets of patients with diabetes and patients with periodontitis with a PPD of ≥4 mm may be useful for screening for type 2 diabetes mellitus. These findings are expected to contribute to the early detection of type 2 diabetes through the dental setting.  The limitation of this study is a small sample size that cannot allow us to current data as a standard for the general population. Although measurement using Bland-Altman, correlation, and regression analyses determined the usefulness of the GBG, however, more accurate measurements need to be confirmed using a large number of participants with a variety of clinical conditions.  This study suggests that GBG may be useful for screening for diabetes in dentistry. In contrast, there may be limitations regarding the degree of concordance with FTBG. Local factors such as inflammatory cytokines in periodontal tissues may influence GBG, and further investigation of these factors may allow GBG to be applied in diagnosing and treating diabetes-related periodontitis. |
| Other information | |  | | |
| Funding | 22 | Give the source of funding and the role of the funders for the present study and, if applicable, for the original study on which the present article is based | 1 | This study was partially supported by the Northern Advancement Center for Science and Technology  (NOASTEC) Foundation (Sapporo, Hokkaido, Japan). The funders had no role in study design, data  collection and analysis, decision to publish, or preparation of the manuscript. There was no additional external funding received for this study. The following grant information was disclosed by the authors: The Development Grants for Medical Institution Needs. |

*Give information separately for cases and controls in case-control studies and, if applicable, for exposed and unexposed groups in cohort and cross-sectional studies.

**Note:** An Explanation and Elaboration article discusses each checklist item and gives methodological background and published examples of transparent reporting. The STROBE checklist is best used in conjunction with this article (freely available on the Web sites of PLoS Medicine at http://www.plosmedicine.org/, Annals of Internal Medicine at http://www.annals.org/, and Epidemiology at http://www.epidem.com/). Information on the STROBE Initiative is available at www.strobe-statement.org.
